# Early Blood Profile of C57BL/6 Mice Exposed to Chronic Unpredictable Stress

**DOI:** 10.3389/fpsyt.2019.00230

**Published:** 2019-04-24

**Authors:** Lindsay T. McDonald, Marcelo F. Lopez, Kristi L. Helke, M.A. McCrackin, James J. Cray, Howard C. Becker, Amanda C. LaRue

**Affiliations:** ^1^Research Services, Ralph H. Johnson Department of Veterans Affairs Medical Center, Charleston, SC, United States; ^2^Department of Pathology and Laboratory Medicine, Medical University of South Carolina, Charleston, SC, United States; ^3^Department of Psychiatry and Behavioral Sciences, Medical University of South Carolina, Charleston, SC, United States; ^4^Charleston Alcohol Research Center, Medical University of South Carolina, Charleston, SC, United States; ^5^Department of Comparative Medicine, Medical University of South Carolina, Charleston, SC, United States; ^6^MUSC/VA Veterinary Diagnostic Laboratory, Medical University of South Carolina, Charleston, SC, United States; ^7^Department of Biomedical Education and Anatomy, Ohio State University, Columbus, OH, United States; ^8^Hollings Cancer Center, Medical University of South Carolina, Charleston, SC, United States

**Keywords:** chronic unpredictable stress, psychological stress, disease, physiological response, C57BL/6, blood, anemia, iron

## Abstract

Physiological responses to psychological stressors are protective in acute fight or flight situations; however, there is increasing evidence suggesting the detrimental impact of chronic psychological stress on disease. Chronic stress has been associated with inflammation, poor prognosis, increased morbidity, and poor outcome in many diseases including atherosclerosis, cancer, and pulmonary disease. Given the systemic impact of stress, and the role of the hematopoietic system as a rapid responder to homeostatic insults, we hypothesized that early blood profile changes and biochemical alterations could be detected in a model of chronic stress. To test this hypothesis, a variation of the chronic unpredictable stress (CUS) model was employed. Following 10 days of CUS, C57BL/6 mice exhibited a chronic-stress-associated corticosterone profile. Complete blood count (CBC) revealed mild normochromic, normocytic anemia, and reduced monocyte and lymphocyte count. Serum analysis demonstrated hypoferremia with unchanged total iron binding capacity and serum ferritin levels. These findings are consistent with clinical diagnostic parameters for anemia of chronic disease and indicate that CUS results in significant changes in blood and serum biochemical profile in C57BL/6 mice. These studies identify early changes in blood parameters in response to CUS and identify hematopoietic and biochemical alterations that are often associated with increased morbidity in patients experiencing chronic-stress-associated mental health disease.

## Introduction

Chronic stress has been associated with chronic illness such as obesity and depression and contributes to worse prognosis, accelerated disease progression, and poorer survival in many diseases including lung pathologies (1, 2), cardiovascular disease (3) [reviewed in Refs. (4, 5)], and cancer [reviewed in Ref. (6)]. In addition to altered mood and depressive or anxious behavior, chronic stress has been linked with increased inflammation (7–9) [reviewed in Refs. (10, 11)], hematopoietic stem cell activation (3), and functional impacts on immune populations (3, 9) and on erythropoiesis (12). Given the significant impact of chronic stress on patient morbidity and mortality, it is important to understand the physiological impact of chronic stress, and to identify ways in which the stress response may contribute to exacerbation of pathologies. Elucidating hematologic changes early after stress may provide insight into mechanisms of disease exacerbation and may allow for early detection of stress-related symptomology and pathology.

Herein, we tested the hypothesis that early blood profile changes and biochemical alterations could be detected in a model of chronic stress. Using a variation of the chronic unpredictable stress (CUS) model, we demonstrated changes in serum corticosterone levels associated with a chronic stress response and dysregulation of the hypothalamic–pituitary–adrenal axis (HPA) often found in patients with depressive mood (13) and anxiety (14) or in caregivers (15). We also demonstrated significant blood profile changes that were readily detected *via* complete blood count (CBC) and serum iron analyses. These studies are significant in that they identify early hematopoietic and biochemical parameters altered by stress in C57BL/6 mice. These findings also provide insight into potential factors that may contribute to chronic psychological stress-induced exacerbation of disease.

## Materials and methods

### Animals

B6.SJL-*Ptprc^a^Pepc^b^*/BoyJ C57BL/6 adult male mice aged 12–16 weeks were bred in-house or were obtained from The Jackson Laboratory (Stock #002014) (Bar Harbor, Maine, USA). Male mice were selected to reduce variability due to the effect of hormones on hematopoiesis. Studies were conducted at the Charleston Veterans Affairs Medical Center (VAMC), an Association for Assessment and Accreditation of Laboratory Animal Care (AAALAC) International-accredited program, with permission and oversight from the Charleston VA Institutional Animal Care and Use Committee (IACUC).

### Chronic Unpredictable Stress Procedures

CUS cohort animals were subjected to each of the following stress procedures in variable order, at a rate of one stress procedure per day as described below, for 10 consecutive days. Stressors were based on existing models of chronic unpredictable/variable stress (3, 16, 17). Stressors included the following: **rapid light/dark cycle** (an automatic light timer was used to expose animals to bright light or darkness in 7-min on/off cycles for 2 h), **cage tilt** (animal cages were placed on a platform at a 45° angle for 6 h with the orientation of the cages rotated 180° hourly), **predator urine exposure** (coyote urine, Wildlife Research Center, Ramsey, MN) (a cotton ball with approximately 100 µl of coyote urine was placed on a petri dish on a wire rack inside a standard mouse cage outside of the reach of the mice for 10 min, with food and water removed for the 10-min duration of the exposure), **reversed light cycle** (animals were exposed to continuous room light for 24 h to enter a reverse light cycle pattern; this was followed by 48 h of a reversed 12-h on/off cycle, lights then remained on for 24 h to reenter a normal 12-h on/off light cycle; this altered light cycle was counted as 2 days of stress procedures), **damp bedding** (animals were transferred to standard home cages with damp corncob bedding and nesting material removed for 6 h), and **rocking** (standard home cages were secured to a plate rocker with forward/backward motion at ∼30 rpm for 2 h). For acute stress data, animals were only subjected to a single predator urine exposure (as described above) 1 h prior to peripheral blood/serum analysis. Nonstressed control animals were untreated. Standard husbandry was maintained throughout the study for both cohorts.

### Peripheral Blood/Serum Analysis

Blood was collected from control and CUS cohort animals 1–3 h following the final stress procedure, between 10 am and 1 pm, on the 10th day. Complete blood count (CBC) was measured by Medical University of South Carolina (MUSC) Department of Comparative Medicine using a Hemavet 950FS Hematology System (Drew Scientific, Miami Lakes, FL). For serum analysis, clotted blood was centrifuged for 10–20 min at 4°C and 13,000 rpm, and serum was isolated. Iron analysis was performed by Cornell University (Cornell University Veterinary Diagnostic Laboratories, Ithaca, NY, USA), Corticosterone quantification was performed according to the manufacturer’s instructions [Corticosterone Enzyme Immunoassay (EIA) Kit, Arbor Assays, Ann Arbor, MI, USA]. Serum ferritin levels were quantified by Enzyme Linked Immunosorbent Assay (ELISA) according to manufacturer’s instructions (Abcam, Cambridge, MA, USA).

### Statistical Analysis

Parametric tests (Student’s *t* tests or ANOVA where appropriate) were used where assumptions of normality (Shapiro–Wilk test) and homogeneity of variance (Brown–Forsythe test) were met. Violations of these assumptions (*p* ≤ 0.05) precipitated implementation of Welch’s corrections, data transformation, or nonparametric alternative testing (Mann–Whitney *U* or Kruskal–Wallis) where appropriate. Data for all experimental replicates were pooled prior to statistical analysis. *p ≤ 0.05 was considered significant for all tests (SPSS 24.0, IBM Corp, Armonk, NY, USA). Figures were generated using GraphPad Prism 5 Software (La Jolla, CA, USA).

## Results

**Figure 1:**
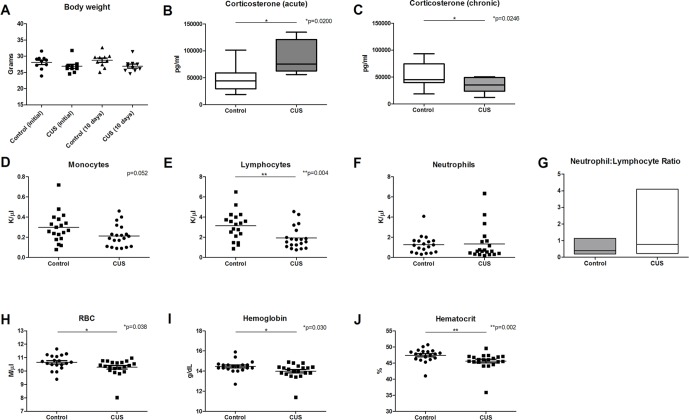
CUS response and CBC analysis. Chronic stress response was measured in animals following 1 day and 10 days of CUS procedures and compared to nonstressed control animals. **(A)** Body weight was measured at 1 day and 10 days following CUS, demonstrating a significant effect of stress [*F*(1,36) = 5.590], but there was no significant interaction between time and stress [*F*(1,36) = 0.259] and there was no significant effect of time [*F*(1,36) = 0.259, *p* = 0.614]. **(B)** Serum corticosterone levels were significantly elevated following acute stress (1 day), [*t*(14) = 2.915, *p = 0.011]. **(C)** Serum corticosterone levels were significantly decreased following 10 days of CUS procedures [*t*(20) = 2.431, *p = 0.025]. CBC comparison including **(D)** monocyte count (MO) [*t*(37) = 2.007, *p* = 0.052], **(E)** lymphocyte count (LY) [*t*(37) = 3.032, **p = 0.004], **(F)** neutrophil count (K/µl), and **(G)** neutrophil (K/µl)-to-lymphocyte (K/µl) ratio (NLR) [*U* = 351, *p* = 0.380; *F*(19,18) = 25.60, *p < 0.001] in control versus CUS cohort animals showed hematopoietic alterations as a result of CUS procedures. **(H)** Red blood cell (RBC) count (*U* = 116.5, *p = 0.038), **(I)** hemoglobin (*U* = 113, *p = 0.030), and **(J)** hematocrit were also reduced in CUS versus control cohort (*U* = 84.5, **p = 0.002). (*p ≤ 0.05, **p ≤ 0.01).

To examine the impact of chronic stress on early blood profile, a variation of the CUS model was employed wherein mice were subjected to one mild psychological or physical stress procedure per day, for 10 days. Stressors were presented in a variable, unpredictable order and were altered daily. As a measure of stress response, body weight (18) was measured at time zero and again at 10 days. There was a significant effect of stress on body weight; however, there was no significant effect of time, and there was no interaction between time and stress (**Figure 1A**).

Serum corticosterone levels are a common measure of stress response. To ensure that animals experience an acute elevation of corticosterone in response to acute stress, a single stressor of predator urine exposure was applied to a cohort of animals (acute stress). Following acute stress, corticosterone levels were significantly elevated (**Figure 1B**, 48.462 ng/ml versus 85.64 ng/ml, *p = 0.011). Serum corticosterone levels were also assessed following 10 days of stress procedures (chronic stress). At 10 days, CUS-exposed mice exhibited significantly lower levels of corticosterone versus control animals (**Figure 1C**, 54.04 ng/ml versus 34.56 ng/ml, respectively, *p = 0.025). This blunted glucocorticoid response is consistent with dysregulation of the HPA axis (19) and is consistent with a chronic stress response (20, 21), adrenal insufficiency (21), or corticosterone-response habituation (22) [reviewed in Ref. (23)].

To test the hypothesis that chronic stress results in early changes in hematologic parameters, peripheral blood was collected from CUS and control cohort animals following 10 days of CUS procedures, and complete blood count (CBC) analysis was performed. Results demonstrated decreased monocyte count (**Figure 1D**, 0.214 K/µl vs. 0.297 K/µl, respectively, *p* = 0.052). CUS cohort animals also exhibited significantly decreased lymphocyte count (**Figure 1E**, 1.939 K/µl vs. 3.134 K/µl, respectively, *p = 0.004) indicating lymphocytopenia. Neutrophil count was increased in the CUS cohort but did not reach statistical significance (**Figure 1F**, 1.261 K/µl vs. 1.338 K/µl, respectively, *p* = 0.5604). The neutrophil-to-lymphocyte ratio (NLR) was also analyzed, demonstrating a trend toward increased NLR in the CUS cohort (**Figure 1G**, 0.803 vs. 0.412, respectively, *p* = 0.380). When the NLR was further investigated to determine if there was an increase in variability due to stress, the variance ratio test revealed a significant increase in variance compared to control (*p < 0.001). An elevated NLR has been accepted as a clinical biomarker of inflammation and a predictor of poor prognosis in disease (24). Increased variance in this ratio reflects individual response to CUS and indicates that an inflammatory state is induced by CUS in C57BL/6 mice. These changes may represent one mechanism by which chronic stress contributes to disease exacerbation.

**Figure 2:**
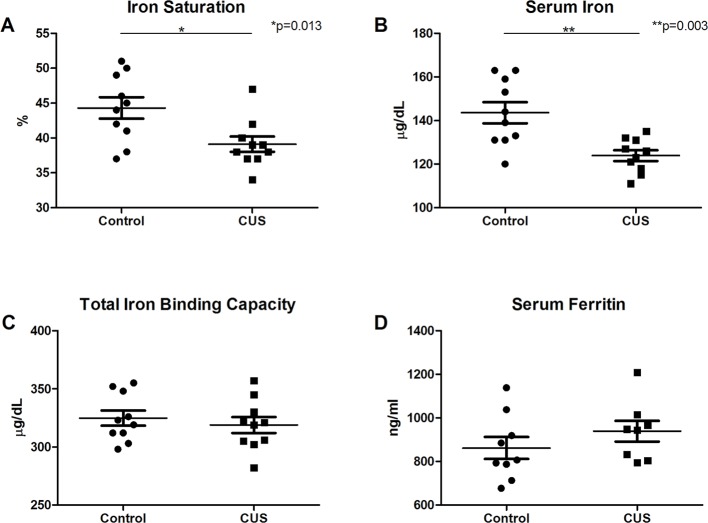
Serum analysis. **(A)** Iron saturation [*t*(18) = 2.753, *p = 0.013] and **(B)** serum iron concentration were decreased in CUS versus control cohort [*t*(13.387) = 3.645, **p = 0.003]. **(C)** Total iron binding capacity was not significantly altered [*t*(18) = 0.625, *p* = 0.540]. **(D)** Serum ferritin levels showed a nonsignificant increase in CUS versus control cohort [*t*(15) = 1.104, *p* = 0.287]. (*p ≤ 0.05, **p ≤ 0.01).

Interestingly, CUS animals exhibited a significant reduction in red blood cell (RBC) count versus control (**Figure 1H**, 10.28 M/µl vs. 10.65 M/µl, respectively, *p = 0.038). RBC parameters were unchanged (data not shown). Hemoglobin was significantly reduced in the CUS cohort [**Figure 1I**, 13.99 g/dL (CUS) vs. 14.45 g/dL (control), *p = 0.030] as were hematocrit levels [**Figure 1J**, 45.64% (CUS) vs. 47.43% (control), **p = 0.002]. Platelet count and mean platelet volume were unchanged (data not shown). Together, this demonstrates mild normocytic, normochromic anemia. To investigate the biological significance of these changes, serum samples were analyzed for iron parameters. Iron saturation was significantly reduced in the CUS cohort (**Figure 2A**, 39.10% vs. 44.30% in control mice, *p = 0.013) as were serum iron levels (**Figure 2B**, 123.9 µg/dL vs. 143.6 µg/dL, respectively, **p = 0.003), indicating hypoferremia resulting from CUS. To exclude iron deficiency anemia, total iron binding capacity (TIBC, a measure of transferrin levels) was examined and was not significantly altered in the CUS cohort [**Figure 2C**, 318.9 µg/dL (CUS) vs. 324.8 µg/dL (control), *p* = 0.540]. Thereby, iron deficiency was excluded as a cause of anemia in CUS cohort animals. Anemia of chronic disease is a chronic condition known to affect patients with chronic illnesses and is diagnostically defined by mild anemia, hypoferremia, normal or increased TIBC, and normal or elevated serum ferritin levels (25). Therefore, serum ferritin levels were quantified, revealing a nonsignificant increase in serum ferritin in the CUS cohort (**Figure 2D**, 939.1 ng/ml vs. 861.9 ng/ml in control mice, *p* = 0.287), consistent with clinical diagnostic parameters for anemia of chronic disease.

## Discussion

This study demonstrates that CUS leads to early and clinically relevant blood profile and biochemical changes in C57BL/6 mice that are readily detected in live animals following 10 days of CUS. While behavioral assessments are often employed to determine the impact of CUS on depressive and anxious response, interpretation of results can be challenging due to strain-specific responses (26), and these changes can take 4–8 weeks to manifest (18), particularly in C57BL/6 mice. In this study, stress response was verified based on corticosterone levels, wherein we demonstrate an acute elevation in corticosterone following exposure to a single stressor and decreased corticosterone response to stress following 10 days of CUS procedures. CUS cohort animals also exhibited neutropenia, lymphocytopenia, as well as a mild decrease in monocyte count and increased variance in NLR, demonstrating immune impacts resulting from chronic stress. Increased NLR (24, 27) and mild anemia have been reported in patient populations as a result of chronic stress or chronic inflammation (28, 29). Also consistent with our findings, a stress-induced decrease in monocytes and T cells in humans has been reported (30). However, other studies have demonstrated elevated monocyte levels in the blood of chronically stressed humans and increased contribution of inflammatory cell populations from bone marrow resulting from chronic stress in mice (3). While our study did not demonstrate these changes in the blood at the early (10-day) time point, it is possible that continued CUS would result in similar changes or that the observed reduction in circulating immune cells may be reflective of redistribution of these cells from the blood to the tissue. Thus, it is important to note that the immune impact of chronic stress can be highly time and context dependent and can vary with intensity and duration of the stressor, the strain or animal model employed, and the method and tissue type examined (30, 31) [reviewed in Ref. (23)].

Iron-related changes, however, have been more consistent between studies and models of psychological stress (2, 12, 32–34). Similarly, our findings demonstrate mild hypoferremia, as well as normocytic normochromic anemia induced by chronic stress. Changes in iron absorption resulting from chronic stress have also been reported (33). While our study did not examine the effect of stress on iron absorption or dietary intake, body weight was not significantly altered between the control and CUS cohort at endpoint, nor was total iron binding capacity suggesting that the hypoferremia identified was not due to iron deficiency. Instead, our findings were consistent with diagnostic parameters for anemia of chronic disease. While the mechanisms driving anemia of chronic disease are not fully understood, glucocorticoids and inflammatory factors such as interleukin-6 (IL-6) and the IL-6–hepcidin axis (35) are believed to be involved. Clinically, anemia of chronic disease leads to shortness of breath, exercise intolerance, and, potentially, organ failure. Thus, iron-related changes such as anemia of chronic disease may represent a novel hematopoietic driver of chronic-stress-induced exacerbation of disease.

There are multiple possible extensions of these findings including in-depth examination of specific impacts on immune populations such as monocyte activation state and analysis of T cell polarization (e.g., Th1/Th2) resulting from CUS. Future studies will examine these possibilities and will assess the longitudinal physiological impact of CUS-induced changes in the context of disease. This study is significant in that it demonstrates important changes in CBC and iron analyses, providing insight into potential novel early diagnostic parameters and hematopoietic changes induced by chronic stress. While additional studies are needed to fully characterize the functional impact of changes in blood and iron parameters, these are important factors that may contribute to disease exacerbation or exacerbation of symptoms in patients with comorbid chronic stress.

## Ethics Statement

This study was carried out in accordance with permission and oversight by the Charleston VA IACUC, an AAALAC International accredited program.

## Author Contributions

Concept and design: LM, HB, and AL; data acquisition: LM, ML, and AL; analysis and data interpretation: LM, ML, KH, MM, JC, and AL; drafting of article/revisions: LM, ML, KH, MM, JC, and AL; final approval: LM, ML, KH, MM, JC, HB, and AL.

## Funding

VAMC MERIT awards to ACL (BX002277 and BX000333), U01 AA014095 (ML and HB), U24 AA020929 (ML and HB), P50 AA010761 (ML and HB), and VA Medical Research. I01BX000813 (HB).

## Conflict of Interest Statement

The authors declare that the research was conducted in the absence of any commercial or financial relationships that could be construed as a potential conflict of interest.

## References

[1] Chen H Liu D Guo L Cheng X Guo N Shi M Chronic psychological stress promotes lung metastatic colonization of circulating breast cancer cells by decorating a pre-metastatic niche through activating beta-adrenergic signaling. J Pathol (2018) 244:49–60. 10.1002/path.4988 28940209

[2] Bible LE Pasupuleti LV Gore AV Sifri ZC Kannan KB Mohr AM Chronic restraint stress after injury and shock is associated with persistent anemia despite prolonged elevation in erythropoietin levels. J Trauma Acute Care Surg (2015) 79:91–6. discussion 96–7. 10.1097/TA.0000000000000686 PMC4902106 26091320

[3] Heidt T Sager HB Courties G Dutta P Iwamoto Y Zaltsman A Chronic variable stress activates hematopoietic stem cells. Nat Med (2014) 20:754–8. 10.1038/nm.3589 PMC4087061 24952646

[4] Golbidi S Frisbee JC Laher I Chronic stress impacts the cardiovascular system: animal models and clinical outcomes. Heart Circ Physiol (2015) 308:H1476–98. 10.1152/ajpheart.00859.2014 25888514

[5] Cohen S Janicki-Deverts D Miller GE Psychological stress and disease. JAMA (2007) 298:1685–7. 10.1001/jama.298.14.1685 17925521

[6] Chida Y Hamer M Wardle J Steptoe A Do stress-related psychosocial factors contribute to cancer incidence and survival? Nature clinical practice. Oncology (2008) 5:466–75. 10.1038/ncponc1134 18493231

[7] Cohen S Janicki-Deverts D Doyle WJ Miller GE Frank E Rabin BS Chronic stress, glucocorticoid receptor resistance, inflammation, and disease risk. Proc Natl Acad Sci U S A (2012) 109:5995–9. 10.1073/pnas.1118355109 PMC3341031 22474371

[8] Miller GE Cohen S Ritchey AK Chronic psychological stress and the regulation of pro-inflammatory cytokines: a glucocorticoid-resistance model. Health Psychol (2002) 21:531–41. 10.1037/0278-6133.21.6.531 12433005

[9] Miller GE Chen E Sze J Marin T Arevalo JM Doll R A functional genomic fingerprint of chronic stress in humans: blunted glucocorticoid and increased NF-kappaB signaling. Biol Psychiatry (2008) 64:266–72. 10.1016/j.biopsych.2008.03.017 PMC2581622 18440494

[10] Chrousos GP Stress, chronic inflammation, and emotional and physical well-being: concurrent effects and chronic sequelae. J Allergy Clin Immunol (2000) 106:S275–91. 10.1067/mai.2000.110163 11080744

[11] Dhabhar FS Effects of stress on immune function: the good, the bad, and the beautiful. Immunol Res (2014) 58:193–210. 10.1007/s12026-014-8517-0 24798553

[12] Vignjevic S Budec M Markovic D Dikic D Mitrovic O Mojsilovic S Chronic psychological stress activates BMP4-dependent extramedullary erythropoiesis. J Cell Mol Med (2014) 18:91–103. 10.1111/jcmm.12167 24283209 PMC3916121

[13] Cheng T Dimitrov S Pruitt C Hong S Glucocorticoid mediated regulation of inflammation in human monocytes is associated with depressive mood and obesity. Psychoneuroendocrinology (2016) 66:195–204. 10.1016/j.psyneuen.2016.01.008 26829709 PMC4792525

[14] Schreiber W Lauer CJ Krumrey K Holsboer F Krieg JC Dysregulation of the hypothalamic-pituitary-adrenocortical system in panic disorder. Neuropsychopharmacology (1996) 15:7–15. 10.1016/0893-133X(95)00146-5 8797187

[15] Miller GE Murphy ML Cashman R Ma R Ma J Arevalo JM Greater inflammatory activity and blunted glucocorticoid signaling in monocytes of chronically stressed caregivers. Brain Behav Immun (2014) 41:191–9. 10.1016/j.bbi.2014.05.016 PMC4973629 25242587

[16] Larsen MH Mikkelsen JD Hay-Schmidt A Sandi C Regulation of brain-derived neurotrophic factor (BDNF) in the chronic unpredictable stress rat model and the effects of chronic antidepressant treatment. J Psychiatr Res (2010) 44:808–16. 10.1016/j.jpsychires.2010.01.005 20172535

[17] Barnum CJ Pace TW Hu F Neigh GN Tansey MG Psychological stress in adolescent and adult mice increases neuroinflammation and attenuates the response to LPS challenge. J Neuroinflammation (2012) 9:9. 10.1186/1742-2094-9-9 22248083 PMC3283491

[18] Monteiro S Roque S de Sa-Calcada D Sousa N Correia-Neves M Cerqueira JJ An efficient chronic unpredictable stress protocol to induce stress-related responses in C57BL/6 mice. Front Psychiatry (2015) 6:6. 10.3389/fpsyt.2015.00006 25698978 PMC4313595

[19] Sorocco KH Lovallo WR Vincent AS Collins FL Blunted hypothalamic-pituitary-adrenocortical axis responsivity to stress in persons with a family history of alcoholism. Int J Psychophysiol (2006) 59:210–7. 10.1016/j.ijpsycho.2005.10.009 PMC2267108 16360227

[20] Gong S Miao YL Jiao GZ Sun MJ Li H Lin J Dynamics and correlation of serum cortisol and corticosterone under different physiological or stressful conditions in mice. PloS One (2015) 10:e0117503. 10.1371/journal.pone.0117503 25699675 PMC4336318

[21] Reber SO Birkeneder L Veenema AH Obermeier F Falk W Straub RH Adrenal insufficiency and colonic inflammation after a novel chronic psycho-social stress paradigm in mice: implications and mechanisms. Endocrinology (2007) 148:670–82. 10.1210/en.2006-0983 17110427

[22] Cole MA Kalman BA Pace TW Topczewski F Lowrey MJ Spencer RL Selective blockade of the mineralocorticoid receptor impairs hypothalamic-pituitary-adrenal axis expression of habituation. J Neuroendocrinol (2000) 12:1034–42. 10.1046/j.1365-2826.2000.00555.x 11012846

[23] Herman JP McKlveen JM Ghosal S Kopp B Wulsin A Makinson R Regulation of the hypothalamic-pituitary-adrenocortical stress response. Compr Physiol (2016) 6:603–21. 10.1002/cphy.c150015 PMC4867107 27065163

[24] Zahorec R Ratio of neutrophil to lymphocyte counts—rapid and simple parameter of systemic inflammation and stress in critically ill. Bratislavske lekarske listy (2001) 102:5–14. 11723675

[25] Lichtman MA Williams WJ Williams hematology. New York: McGraw-Hill, Medical Pub. Division (2006).

[26] Jung YH Hong SI Ma SX Hwang JY Kim JS Lee JH Strain differences in the chronic mild stress animal model of depression and anxiety in mice. Biomol Ther (Seoul) (2014) 22:453–9. 10.4062/biomolther.2014.058 PMC4201223 25414777

[27] Hickman DL Evaluation of the neutrophil:lymphocyte ratio as an indicator of chronic distress in the laboratory mouse. Lab Animal (2017) 46:303–7. 10.1038/laban.1298 PMC7091828 28644453

[28] Zarychanski R Houston DS Anemia of chronic disease: a harmful disorder or an adaptive, beneficial response? Can Med Assoc J (2008) 179:333–7. 10.1503/cmaj.071131 PMC2492976 18695181

[29] Tian R Hou G Li D Yuan TF A possible change process of inflammatory cytokines in the prolonged chronic stress and its ultimate implications for health. ScientificWorldJournal (2014) 2014:780616. 10.1155/2014/780616 24995360 PMC4065693

[30] Maydych V Claus M Dychus N Ebel M Damaschke J Diestel S Impact of chronic and acute academic stress on lymphocyte subsets and monocyte function. PLoS One (2017) 12:e0188108. 10.1371/journal.pone.0188108 29145439 PMC5690587

[31] McKim DB Yin W Wang Y Cole SW Godbout JP Sheridan JF Social stress mobilizes hematopoietic stem cells to establish persistent splenic myelopoiesis. Cell Rep (2018) 25:2552–62 e3. 10.1016/j.celrep.2018.10.102 PMC6342493 30485819

[32] Wang L Wang W Zhao M Ma L Li M Psychological stress induces dysregulation of iron metabolism in rat brain. Neuroscience (2008) 155:24–30. 10.1016/j.neuroscience.2008.03.091 18555617

[33] Chen J Shen H Chen C Wang W Yu S Zhao M The effect of psychological stress on iron absorption in rats. BMC Gastroenterol (2009) 9:83. 10.1186/1471-230X-9-83 19912618 PMC2783024

[34] Wei C Zhou J Huang X Li M Effects of psychological stress on serum iron and erythropoiesis. Int J Hematol (2008) 88:52–6. 10.1007/s12185-008-0105-4 18543064

[35] Fraenkel PG Understanding anemia of chronic disease. Hematology American Society of Hematology Education Program (2015) 2015:14–8. 10.1182/asheducation-2015.1.14 26637695

